# Practicing What They Preach: Do Medical Specialty and Subspecialty Conferences Incorporate Physician Wellness Into Their Programs?

**DOI:** 10.7759/cureus.76688

**Published:** 2024-12-31

**Authors:** Nicole M Mahr, Daryl H Thorne, Andrew S Thagard

**Affiliations:** 1 Obstetrics and Gynecology, F. Edward Hebert School of Medicine, Uniformed Services University, Bethesda, USA; 2 Family Medicine, F. Edward Hebert School of Medicine, Uniformed Services University, Bethesda, USA

**Keywords:** medical conferences, physicians, resilience, well-being, wellness, wellness domains

## Abstract

Background: Depending upon their organization and content, medical conferences can enhance wellness or create additional stress for physician attendees. The objective of our study was to examine the degree to which major medical specialty and subspecialty conferences incorporate wellness into their meeting programs.

Methods: We performed a cross-sectional study of major medical conferences occurring in 2023 representing specialties and subspecialties with the greatest number of active physicians. Meeting programs and agendas were reviewed for conference timing including if scheduled on a weekend and/or United States Federal or major religious holiday, availability of childcare, and wellness-themed activities and sessions. Sessions were categorized across seven wellness domains, including emotional, environmental, financial, occupational, physical, social, and spiritual.

Results: Programing was available for 39 of the 41 conferences. The median conference length was five days. Approximately 97% (38/39) of conferences were held over all or part of a weekend and 21% (8/39) were scheduled over a Federal or religious holiday. Only one in 10 meetings advertised childcare. Fifty-four percent of conferences included a wellness activity such as a fun run, yoga, meditation, or city tour. The majority of agendas included sessions across multiple wellness domains, most commonly social (n=427), occupational (n=356), and emotional (n=140).

Conclusion: Most major medical specialty and subspecialty conferences incorporate wellness-themed activities and sessions into their agendas; however, opportunities exist to improve the availability of childcare, optimize scheduling, and offer more expansive programing across wellness domains.

## Introduction

Neglecting personal wellness among physicians remains a significant concern across specialties and affects the individual clinician, healthcare costs, and patient care [1-6]. A shift in the clinical practice environment from majority small-group or solo practice to employment by large healthcare organizations has exacerbated the problem [7].

The solution to prioritizing wellness among physicians is complex with no single solution. Proposals include proactive engagement and self-calibration, building community with colleagues, and engaging in activities to enhance self-awareness [3-6]. Medical associations and professional organizations can serve as a means of enhancing connection, collaboration, and the exchange of knowledge [5,6].

Annual conferences sponsored by medical specialties create an opportunity to promote physician well-being and fulfillment. Cited advantages of conference attendance include building interpersonal relationships and professional development [8,9]. A 2023 Association of University Radiologists meeting incorporated art, music, and experiential wellness activities designed to foster connection and community. Post-meeting survey data suggested attendees left with an improved meeting experience and tools for effective stress management [10]. However, specialty meetings that separate physicians from their families, occur on weekends or holidays or are over-scheduled with dense information sessions may increase, rather than mitigate, attendee stress. The purpose of our study was to evaluate the degree to which wellness concepts are currently integrated into the programing of major specialty conferences.

This study was presented as a poster at the American College of Obstetricians and Gynecologists Armed Forces District Annual Meeting in October 2024.

## Materials and methods

We performed a cross-sectional study to review the programing of medical specialty conferences in 2023. The study protocol was reviewed and exempted by the Naval Medical Center Portsmouth Institutional Review Board (Portsmouth, Virginia, USA).

Our sample consisted of a major conference for the top medical specialties and subspecialties as determined by the Association of American Medical Colleges [11]. We excluded Pediatric Hematology/Oncology, Pediatric Critical Care Medicine, Pediatric Cardiology, Pediatric Anesthesiology, Internal Medicine/Pediatrics, Neonatal-Perinatal Medicine, and Sports Medicine due to significant overlap with other specialties or subspecialties.

We performed an online search to obtain the meeting agendas. For programs no longer available online, the conference organizers or sponsoring organizations were contacted. If no response was obtained, additional requests were made to these organizations on two separate occasions.

Meeting dates were reviewed to determine if the conference was scheduled over a weekend or a Federal or major religious holiday. We defined major religious holidays according to the five religions in the United States with the largest number of adherents as evaluated by the Pew Research Center’s 2014 Religious Landscape Study: Christianity, Judaism, Islam, Hinduism, and Buddhism [12]. We cross-referenced these to publications from university offices of Spiritual and Religious Life to identify the major holidays most often allotted as excused absences, which we then pared down to emphasize holy days and fasting such as Ash Wednesday, Passover, Rosh Hashanah, Ramadan, and Diwali.

Conferences were classified as advertising onsite childcare if it was mentioned in the meeting agenda. Unscheduled blocks or breaks longer than two hours were counted as “white space.” The program content was then independently reviewed by two investigators for the type and quantity of wellness-specific sessions. Wellness-specific sessions were categorized into seven wellness domains: emotional, environmental, financial, occupational, physical, social, and spiritual [13]. We included invitation-only and ticketed wellness sessions in our analysis but excluded industry-sponsored sessions. Identical sessions that occurred more than once or sessions given as a multi-day series were counted as one session. Sessions that aligned with more than one wellness domain were counted in the tally of its most prominent domain. Session inclusion and categorization were compared between two investigators; any discrepancies were resolved through consensus among all three investigators. Wellness activities were tallied separately.

Data analysis for this observational study consisted of descriptive statistics. Results were reported as number, percentage, and median with standard deviation and/or range when appropriate.

## Results

Program content was available for 39 of 41 meetings. The median conference length was five days with a range of three to seven. Thirty-eight (97%) occurred over part or all of a weekend and one in five were scheduled on a Federal or religious holiday. Affected holidays included Columbus/Indigenous Peoples Day (n=1), the Lunar New Year (n=1), Ramadan (n=4), Eid al-Fitr (n=1), and Diwali (n=1). One of the four conferences scheduled over Ramadan mentioned a prayer or quiet room in their program. Approximately one in 10 (n=4; 10.2%) meetings included mention of onsite childcare in their agendas, and approximately 15% (n=6) built free time or “white space” into their programs. Table 1 provides additional information for individual conferences.

**Table 1:** Individual conference data by specialty

Specialty	Conference	Duration (days)	Scheduled on weekend?	Scheduled on holiday?	Childcare advertised
Allergy and immunology	American Academy of Allergy, Asthma, and Immunology	4	Yes	No	Yes
Anesthesiology	American Society of Anesthesiologists	6	Yes	No	No
Cardiovascular disease	American College of Cardiology	3	Yes	No	No
Child and adolescent psychiatry	American Academy of Child and Adolescent Psychiatry	6	Yes	No	No
Clinical cardiac electrophysiology	Heart Rhythm Society	3	Yes	No	No
Critical care medicine	Society of Critical Care Medicine	6	Yes	Yes (Lunar New Year)	No
Dermatology	American Academy of Dermatology	6	Yes	No	No
Emergency medicine	American College of Emergency Physicians	7	Yes	Yes (Columbus Day)	No
Endocrinology, diabetes, and metabolism	American Association of Clinical Endocrinologists	3	Yes	No	Yes
Gastroenterology	American College of Gastroenterology	6	Yes	No	No
General surgery	American College of Surgeons	4	Yes	No	Yes
Geriatric medicine	American Geriatrics Society	4	Yes	No	No
Hematology and oncology	American Society of Hematology	4	Yes	No	Yes
Infectious disease	Infectious Diseases Society of America	6	Yes	No	No
Internal medicine	American College of Physicians	3	Yes	Yes (Ramadan)	No
Interventional cardiology	Society for Cardiovascular Angiography and Interventions	3	Yes	No	No
Nephrology	American Society of Nephrology	4	Yes	No	No
Neurological surgery	American Association of Neurological Surgeons	5	Yes	Yes (Ramadan)	No
Neurology	American Academy of Neurology	6	Yes	Yes (Eid al-Fitr)	No
Neuroradiology	American Society of Neuroradiology	5	Yes	No	No
Obstetrics and gynecology	American College of Obstetrics and Gynecology	3	Yes	No	No
Orthopedic surgery	American Association of Orthopedic Surgeons	5	Yes	No	No
Ophthalmology	American College of Ophthalmology	4	Yes	No	No
Otolaryngology	American Academy of Otolaryngology - Head and Neck Surgery	5	Yes	No	No
Pain medicine and pain management	American Academy of Pain Medicine	4	Yes	Yes (Ramadan)	No
Pediatrics	American Academy of Pediatrics	5	Yes	No	No
Plastic surgery	American Association of Plastic Surgeons	4	Yes	No	No
Physical medicine & rehabilitation	American Academy of Physical Medicine & Rehabilitation	6	Yes	No	No
Preventive medicine	American College of Preventive Medicine	5	Yes	No	No
Psychiatry	American Psychiatric Association	5	Yes	No	No
Pulmonary disease	American Thoracic Society	6	Yes	No	No
Radiation oncology	American Society for Radiation Oncology	5	Yes	No	No
Radiology and diagnostic radiology	Radiologic Society of North America	5	Yes	No	No
Rheumatology	American College of Rheumatology	6	Yes	Yes (Diwali)	No
Sports medicine	American College of Sports Medicine	4	No	No	No
Thoracic surgery	American College for Thoracic Surgery	4	Yes	No	No
Urology	American Urologic Association	4	Yes	No	No
Vascular and interventional radiology	Society of Interventional Radiologists	5	Yes	Yes (Ramadan)	No
Vascular surgery	Society for Vascular Surgery	5	Yes	No	No

Fifty-four percent of conferences included at least one wellness activity such as a fun run, poetry workshop, community service project, or city tour. Of those that featured wellness activities, the median number of activities was two (SD=6, range 1-25). Less than half of surgical specialty/subspecialty conferences (4/10; 40%) provided at least one wellness activity (range: 1-4 activities): the American College of Surgeons (n=4), American Association of Neurological Surgeons (n=1), American Academy of Otolaryngology/Head and Neck Surgery (n=2), and American Association of Plastic Surgeons (n=2). In comparison, 17 of the 29 medicine specialty/subspecialty conferences (58.6%) provided at least one wellness activity (range: 1- 25 activities). Three of these 17 offered more than 10: the American Academy of Child and Adolescent Psychiatry (n=25), American Academy of Neurology (n=11), and Radiologic Society of North America (n=15).

Most agendas included sessions across multiple wellness domains. The most commonly represented domains were social (n=427), occupational (n=356), and emotional (n=140). Only four conferences provided fewer than 10 wellness content sessions, including the American Association of Plastic Surgeons, American Academy of Pain Medicine, American Society of Nephrology, and Heart Rhythm Society. The American Academy of Neurology provided the most opportunities at 108 wellness-specific sessions over six days. Overall, surgical specialty/subspecialty conferences (n=10) totaled 268 sessions/activities (mean=26.8 sessions per conference) while medicine specialty/subspecialty conferences (n=29) totaled 985 sessions/activities (mean=33.6 sessions per conference). Table 2 provides examples of sessions aligning with the domains.

**Table 2:** Wellness domains with representative examples

Domain	Description	Number of sessions across all conferences	Conference examples
Emotional	Expressing feelings, coping with challenges	140	“Divorce: What every surgeon should know” (American College of Surgeons) “How failure helps us succeed” (American Academy of Neurology)
Environmental	Creating a culture of safety in the workplace	73	“Prioritizing inclusion, diversity, access and equity (IDA&E) in infectious disease trainee and faculty recruitment” (Infectious Diseases Society of America) “Optimizing the clinical environment: Learning and practicing with intent and inclusion” (Society of Vascular Surgeons)
Financial	Building financial resources, knowledge about personal finance	91	“Divide and conquer: Student loan management plan” (American Society of Neuroradiology) “Negotiating salary in 2023” (American College of Obstetrics and Gynecology)
Occupational	Participating in activities to build occupational network, job satisfaction	359	“Switching jobs: When to know it’s time” (Society for Cardiovascular Angiography) “Mentorship: Nuts and bolts of the gift of a dynamic reciprocal relationship” (American Psychiatric Association)
Physical	Fitness, nutrition, health maintenance	42	“Ergonomics in the clinic and OR” (American College of Ophthalmology) “Culinary medicine: Using food for physical, mental, and environmental health” (American Academy of Pediatrics)
Social	Building relationships, community	432	“ASH-a-Poolza: Blood wars - Jeopardy!” (Society of Hematology) “RSNA after dark” (Radiologic Society of North America)
Spiritual	Having meaning and purpose	17	“Humanism in medicine: Re-emphasis on human-to-human interactions” (American College of Physicians) “Finding joy in different career stages” (American Academy of Physical Medicine & Rehabilitation)

## Discussion

This cross-sectional study explored the degree to which major medical specialty and subspecialty conferences incorporate wellness and wellness themes into scheduling and programing. Our results demonstrate that over half of meetings included at least one wellness activity and most featured sessions across one or more of the major wellness domains. Despite the majority scheduled over all or part of a weekend and one in five occurring over a holiday, few advertised the availability of onsite childcare. Additionally, few meetings built longer breaks or downtime into their programs.

While the social, occupational, and emotional wellness domains were well represented with over 100 individual sessions each across all specialty conferences, others such as environmental, financial, and physical were incorporated into far fewer activities. In particular, survey data identifies deficiencies in financial literacy among physicians in a variety of specialties suggesting a need for additional tools in this domain [14-16].

Our study has several strengths and some limitations. The scope of our analysis was broad, including one major conference from the specialties and subspecialties with the largest number of active physicians. We obtained programs from the vast majority of our sample (>95%), explored granular data including wellness-specific activities, and evaluated individual sessions across wellness domains. The results provide insight into how medical conferences - frequently attended but uncommonly studied - currently prioritize wellness for their attendees.

In recent years, the value of medical conferences has come under scrutiny. Attendees have questioned the environmental impact, scientific rigor, and influence of industry on these meetings and whether the effort justifies the time and financial cost [17]. Measuring the benefit of these events is challenging and can include, for example, hours of continuing medical education earned or publication rates of meeting presentations [18-20]. Assessing how medical conferences incorporate wellness and wellness themes into their agendas may provide an additional means to assess their value.

Our study is limited in that it provides a single snapshot of medical conferences in one calendar year. Evaluating wellness content over time could offer insight into emerging trends; however, availability of past content is limited and disruptions to in-person meetings prior to 2023 due to the COVID-19 pandemic make comparisons difficult. Our data were obtained from agendas and not by attending individual conferences. As a result, it may not fully capture unscheduled or impromptu wellness activities and other less tangible wellness benefits. Finally, our research does not include the individual voices of attendees or conference organizers. Future qualitative research should explore attendees’ perceptions of wellness incorporation at scientific meetings and differences in attitudes among specialties.

## Conclusions

In summary, our analysis suggests that most major medical societies are including wellness content in their conference programing. We found that nearly all of the major specialty conferences offered sessions encompassing the major wellness domains: emotional, environmental, financial, occupational, physical, social, and/or spiritual health. Additionally, many incorporated wellness activities like sunrise walks, leisure book clubs, live music, and wine tasting. Our results also suggest that opportunities exist to improve access to childcare and designated prayer/quiet spaces, optimize scheduling, and offer more expansive holistic programing across major wellness domains.
